# Treatment with interleukin-2 in malignant pleural mesothelioma: immunological and angiogenetic assessment and prognostic impact

**DOI:** 10.1038/sj.bjc.6605438

**Published:** 2009-11-24

**Authors:** G Alì, L Boldrini, M Lucchi, A Picchi, M Dell'Omodarme, M C Prati, A Mussi, V Corsi, G Fontanini

**Affiliations:** 1 Division of Pathological Anatomy, Department of Surgery, University of Pisa, Via Roma 57, Pisa 56126, Italy; 2 Division of Thoracic Surgery, Department of Cardio-Thoracic Surgery, University of Pisa, Via Paradisa, Pisa 5614, Italy; 3 Scuola Normale Superiore and Istituto di Fisica Nucleare, Section of Pisa, Pisa 56126, Italy

**Keywords:** malignant pleural mesothelioma, tumour microenvironment, interleukin-2, prognosis

## Abstract

**Background:**

Administration of interleukin-2 (IL-2) has shown some effects on malignant pleural mesothelioma (MPM) tumour regression. The purpose of this study was to investigate the ability of IL-2 to modify immunological effector cells and angiogenesis in MPM patients and their prognostic value.

**Methods:**

Tumour-infiltrating lymphocytes (CD4, CD8, Foxp3), mast cells (MCs) (tryptase and chymase), microvessel count (MVC) and VEGF were determined by immunohistochemistry in two series of MPM patients: 60 patients treated with intra-pleural preoperative IL-2 and 33 patients untreated.

**Results:**

Tryptase MCs, and CD8 and Foxp3 lymphocytes were significantly increased in the IL-2-treated group, whereas MVC was significantly lower in the same group. Moreover, in the IL-2-treated group, greater tryptase+MCs and greater Foxp3 lymphocytes were associated with improved and poorer clinical outcomes, respectively. Notably, when these two immunological parameters were combined, they predicted outcomes more effectively.

**Conclusions:**

This study showed that IL-2 treatment leads to a significant increase of immunological parameters, concomitantly with a reduction in vasculature, providing new insight into the cancer mechanisms mediated by IL-2. Moreover, these results suggest that tryptase-positive MCs and Foxp3+ lymphocytes predict clinical outcomes in IL-2-treated patients, highlighting the critical role of the inflammatory response in mesothelioma cancer progression.

Malignant pleural mesothelioma (MPM) is a relatively rare tumour with a growing occurrence in the past few decades throughout the world. It is a fatal neoplasm, with a median survival of 12 months in patients receiving palliative care (Robinson *et al*, 2005). Presently, there is no satisfactory treatment of MPM, although recent reports suggest that multimodality therapy, including surgery, may result in a significant improvement in the survival of select patients (Rusch *et al*, 2001; Lucchi *et al*, 2007).

The precise relationship between the immune system and cancer is still unknown. The tumour–host interaction has a pivotal role in tumour progression and involves various cell types present in the tumour microenvironment, including neoplastic cells, immune effector cells, stromal cells and extracellular matrix components (Zou, 2005). The tumour inflammatory infiltrate is composed of different immune effector cells, such as lymphocytes, macrophages, dendritic cells, and mast cells (MCs) (Balkwill and Mantovani, 2001). Particularly, tumour-infiltrating lymphocytes (TILs) present in the tumour microenvironment have been recognised as principal effectors in modulating tumour immunity (Curiel *et al*, 2004; Yu and Fu, 2006).

Various studies have shown mesothelioma to be sensitive to immunotherapy using cytokines in an attempt to stimulate the anti-tumoural immune response (Robinson *et al*, 2005).

Interleukin-2 (IL-2) was first described as a pro-inflammatory cytokine produced by activated T cells whose main action is to promote proliferation, survival and differentiation of T cells (Cantrell and Smith, 1984; Rubin, 1993; Akbar *et al*, 1996; Armstrong *et al*, 2001). Intravenous, subcutaneous or intra-pleural administration of IL-2 has shown some effects on tumour regression in MPM, supporting the use of IL-2 in clinical trial settings (Goey *et al*, 1995; Astoul *et al*, 1998; Castagneto *et al*, 2001; Mulatero *et al*, 2001; Lucchi *et al*, 2007). However, the mechanism underlying the biological effects of IL-2 on tumour growth is complex and it is still largely unknown.

Interleukin-2 can affect many different cell types, particularly cytotoxic CD8+ T lymphocytes and regulatory T (Treg) cells. The latter cells are a subset of CD4+ T lymphocytes defined by their constitutive expression of the IL-2 receptor *α* chain CD25 (Sakaguchi *et al*, 1996). The nuclei of these cells also contain the Foxp3 transcription factor that is reported to be a key regulatory gene for the development and function of Treg cells and the most specific marker of this type of cells (Hori *et al*, 2003; Fontenot and Rudensky, 2005). Moreover, IL-2 may affect different cell types, including CD4+ T cells, NK cells, macrophages and B cells, resulting in anti-tumour immunity (Jackaman *et al*, 2003). Interleukin-2 could also activate MCs (Maggiano *et al*, 1990; Galli *et al*, 1993), and induce tumour rejection, inhibiting tumour-associated vascularity (Jackaman *et al*, 2003).

The purpose of this study was to evaluate the effect of IL-2 in the MPM microenvironment and to examine the prognostic value of changes in immunological effector cells and angiogenesis in the group of 60 MPM patients who received preoperative IL-2 treatment.

## Materials and methods

### Patients

A total of 93 patients with MPM were included in this study. The specimens were obtained from patients who underwent surgical resection at the Department of Cardio-Thoracic Surgery of the University of Pisa, from January 1999 to May 2008. Participation in this study required informed consent. No patient had received chemotherapy or radiotherapy before surgery. The patients were divided into two groups: patients who received treatment with preoperative intra-pleural IL-2 and untreated patients. Sixty patients, 51 males and 9 females received preoperative immunotherapy with intra-pleural IL-2 (18 × 106 UI per day every other day for three times), according to eligibility criteria and the treatment protocol described earlier (Lucchi *et al*, 2007). After 1 day of recovery, the patients underwent a thoracotomy. Of the 60 patients, 53 (88.3%) had a pleurectomy/decortication (P/D), whereas the remaining 7 patients (11.7%) had an extrapleural pneumonectomy. Thirty-three patients, 27 men and 6 women underwent surgical resection without preoperative immunotherapy with IL-2 or any other preoperative therapy. All of the 33 patients had a P/D.

### Follow-up of IL-2-treated MPM patients

Sixty patients were observed until death or the final date of analysis (August 2008), with a median follow-up time of 74 months for living patients (range, 2–113 months). Forty-one (68.3%) patients relapsed during follow-up: 33 had a local relapse, 7 had both a local and a systemic relapse, and only 1 patient had a systemic relapse. At the end of the follow-up period, 15 patients (25%) were still alive and 45 (75%) had died. Time to progression and overall survival rates were calculated as the period from surgery until the date the disease started to worsen (local and/or systemic recurrences), or date of death (Table 1).

### Tumour specimens

All of the tumour samples were formalin-fixed and paraffin-embedded for microscopic examination. The most representative paraffin block of tumour was selected for immunohistochemical analysis. A histological and pathological diagnosis were reviewed by two pathologists (G Alì and G Fontanini), according to the WHO 2004 histological and immunohistochemical criteria (Travis *et al*, 2004). Disagreements concerning the histological diagnosis were discussed, and after a critical discussion, a mutual agreement was reached. The surgical–pathological staging was performed according to the TNM classification by the International Mesothelioma Interest Group (IMIG) (Rusch, 1995).

### Immunohistochemistry

Immunohistochemical analyses were performed on 3 *μ*m tissue sections using specific antibodies. Immunoreaction was displayed using the avidin–biotin–peroxidase complex method. Peroxidase activity was visualised with diaminobenzidine. Counterstaining was performed with haematoxylin. The negative controls were carried out by omitting the primary antibodies. Immunostaining was done using a Benchmark immunostainer (Ventana, Tucson, AZ, USA). In all cases, the immunohistochemical evaluation was performed independently by two pathologists (G Alì and G Fontanini) who were blind to the clinicopathological characteristics and treatment of the patients.

**Figure 1:**
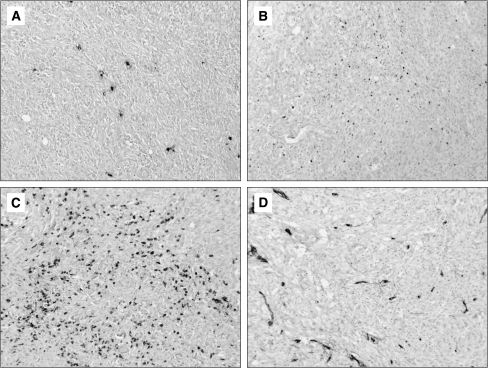
Immunohistochemical staining for tryptase-positive mast cells (**A**), Foxp3-positive lymphocytes (**B**), CD8-positive lymphocytes (**C**), and microvessel count (**D**) in mesotheliomas. Original magnification, × 100.

For the tryptase and chymase immunohistochemical stainings, sections were incubated with a mouse anti-human tryptase monoclonal antibody (Chemicon International, Temecula, CA, USA), used at a 1 : 1500 dilution, and with a mouse anti-human chymase monoclonal antibody (Chemicon International), used at a 1 : 1600 dilution. Immunostaining for tryptase and chymase was clearly visible as brown deposits within intact MCs and as highly localised extracellular granular material (Figure 1A). Each pathologist identified, under low microscopic power ( × 10 objective lens and × 10 ocular lens), the areas where the MCs were the most intensely accumulated, and then counted positive MCs at × 200 microscopic fields. The average of their counts in three fields was calculated as described earlier (Ibaraki *et al*, 2005).

For TIL-immunohistochemical staining, sections were incubated with the following antibodies: mouse anti-human Foxp3 monoclonal antibody (clone 236A/E7 diluted 1 : 300; Abcam, Cambridge, UK) (Figure 1B), mouse anti-human CD8 monoclonal antibody (clone C8/144B, ready to use for the Ventana automated slide stainer; Ventana) (Figure 1C), and mouse anti-human CD4 monoclonal antibody (clone 1F6 diluted 1 : 20; Diagnostic BioSystem, Pleasanton, CA, USA). Each pathologist identified, under low microscopic power ( × 25 objective lens and × 10 ocular lens), the areas where the positive cells were most intensely accumulated, and then counted the cells at × 400 microscopic fields. The average of their counts in five fields was calculated as described earlier (Mizukami *et al*, 2008; Yoshioka *et al*, 2008).

The microvessel count (MVC) was determined using the anti-CD34 antibody (Ventana Medical System, ready to use for the Ventana automated slide stainer) (Figure 1D). Each pathologist examined the samples and identified the area with the most intense vascularisation (hot spot) under low microscopic power ( × 10 objective lens and × 10 ocular lens). In this area, the number of microvessels were counted and recorded at × 400 ( × 40 objective lens and × 10 ocular lens).

For the VEGF expression, the sections were incubated with an anti-VEGF rabbit polyclonal antibody (Santa Cruz Biotechnology, Santa Cruz, CA, USA) used at a 1 : 50 dilution. The expression of VEGF was evaluated as a percentage of positive cells in a total of at least 1000 tumour cells (Ciardiello *et al*, 2001).

### Statistical analysis

Statistical analysis required the use of the following tests: a Student's *t-*test for the analysis of the differences between preoperative IL-2-treated patients *vs* untreated patients; an ANOVA to study the association between risk factors and inflammatory and angiogenetic variables; a Kaplan–Meier test to estimate survival function, and a Cox proportional-hazard model for multivariate survival analysis. The analysis was conducted using R 2.9.1. The level of significance was set at a *P*-value of less than 0.05.

## Results

### Clinicopathological characteristics

Ninety-three patients were enroled in this study. On the basis of histological features, the tumours were classified as epithelioid in 46 patients (76.7%), biphasic in 9 patients (15%) and sarcomatoid in 5 patients (8.3%) in the group of patients treated with preoperative IL-2. Preoperative intra-pleural IL-2 was administered to all of the patients without a dose reduction or interruption. All of the patients experienced a fever during treatment and required antipyretic medication (acetaminophen). Out of the 33 mesotheliomas belonging to the group of untreated patients, 25 (75.8%) were epithelioid, 3 (9%) were biphasic and 5 (15.2%) were sarcomatoid. Other clinicopathological characteristics of the series of patients of both groups are summarised in Table 1.

### Analysis of immunological and angiogenetic parameters between the two groups of treatment

The differences between the two treatment's groups, evaluated by the Student's *t*-test, are summarised in Table 2.

Regarding the MCs, the IL-2-untreated group showed a significantly lower number of the tryptase MCs in comparison with treated patients’ group (*P*=0.04). No differences between the two groups of patients were observed for the chymase MCs.

Immunohistochemistry revealed an increase in the number of CD8+ lymphocytes in the preoperative-treated group in comparison with the untreated patients (*P*=0.004). Regarding the CD4+ lymphocytes, the mean value was higher in the group treated with IL-2 than in the untreated group, but the difference was not statistically significant. Evaluation of Foxp3+ lymphocytes revealed a significant higher number in the treated group compared with the untreated group (*P*=0.02).

As regards MVC, the number of microvessels was significantly lower in the IL-2-treated patients as opposed to untreated patients (*P*=0.0001). On the contrary, no differences between the two groups of patients were observed for the percentage of VEGF+ neoplastic cells.

### Associations between immunological and angiogenetic parameters and clinicopathologic characteristics

Correlations between the analysed parameters and the clinicopathologic characteristics of patients are summarised in Table 3. The MVC was significantly associated with the histologic subtypes of mesothelioma (*P=*0.0047), with a higher MVC for sarcomatoid and biphasic subtypes than for the epithelioid subtype. No other significant associations were found between the immunological and angiogenetic parameters and the clinicopathological parameters.

### Prognostic factors in IL-2-treated MPM patients groups

In univariate analyses, the histologic subtypes were significantly associated with overall and disease-free survival. The sarcomatoid subtype showed a worse overall survival (*P*=0.02) and a shorter time-to-progression (*P*=0.02). No significant association was found between survival and other clinicopathologic parameters (Table 4).

**Figure 2:**
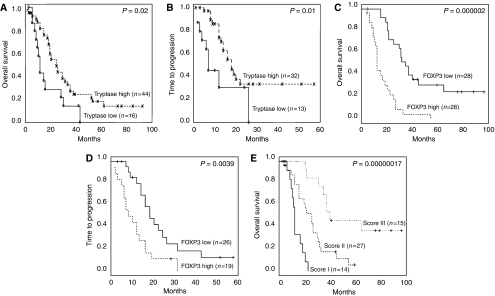
Kaplan–Meier overall survival (**A**) and time to progression (**B**) curves for tryptase MCs counts divided into two groups above and below the median value; Kaplan–Meier overall survival (**C**) and time to progression (**D**) curves for Foxp3 counts divided into two groups above and below the median value; (**E**) Kaplan–Meier overall survival curves for combination of tryptase MCs and Foxp3 counts.

Tryptase-positive MC counts were analysed as a dichotomous variable using the median value to distinguish low (⩽15) from high (>15) MC counts. A statistically significant association was found between the tryptase-positive MC counts and both overall survival (*P=*0.02) and time-to-progression (*P=*0.01) (Table 4). A higher tryptase-positive MC count was significantly associated with a better overall survival and a longer time-to-progression (Figure 2A and B).

The Foxp3 count was also analysed as a dichotomous variable using the median value to distinguish tumours with a low or negative Foxp3 count (⩽8.2) and tumours with a high Foxp3 count (>8.2). Univariate analysis showed that the number of Foxp3+ cells was significantly correlated with overall survival (*P=*0.000002) and time-to-progression (*P=*0.0039) (Table 4). In fact, high numbers of Foxp3+ lymphocytes were unfavourable to both survival and recurrence (Figure 2C and D).

To assess whether tryptase-positive MC and Foxp3+ lymphocyte counts in the tumours were independent predictors of survival, a Cox proportional-hazard model was used to carry out multivariate analysis. After stratifying for gender, age, histology, performance status and stage, the two variables maintained their independent prognostic roles (Table 5).

### Combination of tryptase-positive MC and Foxp3+ lymphocyte density

The prognostic influence of the combination of tryptase-positive MCs and Foxp3+ lymphocytes was then evaluated. Patients were classified into three groups: I (*n*=14), Foxp3+ lymphocytes high and tryptase-positive MCs low; II (*n*=27), both high density or both low density; and III (*n*=15), Foxp3+ lymphocytes low and tryptase-positive MCs high. Significant differences in overall survival were found among the three groups (*P=*0.00000017) (Figure 2E). Group III has better overall survival than groups II and I.

## Discussion

The tumour–host interaction involves various cell types present in the tumour microenvironment and this interaction could have contradictory effects on tumour progression by either supporting tumour growth or by preventing tumour development (Coussens and Werb, 2002; Mantovani *et al*, 2008).

IL-2 is a cytokine described to promote activated T-cell proliferation, survival and differentiation (Cantrell and Smith, 1984; Rubin, 1993; Akbar *et al*, 1996; Armstrong *et al*, 2001). Reduced levels of IL-2 have been associated with a lower survival of metastatic cancer patients (Herberman, 1984; Lissoni *et al*, 1991), and numerous trials have been performed using IL-2 alone or in combination with other forms of immunotherapy and chemotherapy (Grande *et al*, 2006). Several studies have shown an anti-tumoural activity of intra-pleural administration of IL-2 for the treatment of MPM, supporting the use of IL-2 in clinical trials (Goey *et al*, 1995; Astoul *et al*, 1998; Castagneto *et al*, 2001; Mulatero *et al*, 2001; Lucchi *et al*, 2007). However, the mechanism underlying the effects of IL-2 in the modification of the MPM microenvironment is still unknown.

In this study, we observed a significant increase of tryptase MCs in IL-2 preoperative-treated patients compared with untreated patients. In a previous study, Maggiano *et al* (1990) suggested that human MCs express IL-2 receptors on their surface. Furthermore, MCs are known to produce and release different cytokines, including IL-2, promoting the recruitment of immune effector cells (Galli *et al*, 1993). Other studies (Panelli *et al*, 2002; Den Otter *et al*, 2008) suggest that the recruitment of immune cells induced by IL-2 administration at the tumour site could be also because of an indirect affect of IL-2 through MCs activation.

Regarding the TILs, we observed a statistically significant increase in both CD8 and Foxp3 lymphocytes in the IL-2 treatment group. The number of CD4 cells was also increased in the same group, but this finding was not statistically significant. A recent study evaluated various lymphocyte subpopulations after intra-tumoural administration of IL-2 in a murine model of mesothelioma (Jackaman *et al*, 2003). The authors showed that IL-2 administration inhibited tumour growth and prolonged survival and that both CD8 and CD4 lymphocytes were required for tumour regression. CD8 T-cells are known have a pivotal role in mediating local tumour immunity throughout antigen-specific tumour cell killing (Zou, 2006). These findings provide a rationale for using IL-2 to enhance CD8+cytotoxic lymphocyte activity to improve tumoural regression.

Regulatory T (Treg) cells, a subset of CD4+CD25+Foxp3+ T lymphocytes, are mediators with the functional ability to regulate/suppress tumour immunity (Zou, 2006). High levels of Treg lymphocytes have been observed in malignant tumours (Chattopadhyay *et al*, 2005) compared to benign lesions and normal tissue (Liyanage *et al*, 2002; Ichihara *et al*, 2003; Curiel *et al*, 2004). In a murine model of mesothelioma (Needham *et al*., 2006), intra-tumoural recruitment of Treg lymphocytes constituted an immune evasion mechanism leading to an increase in tumour growth. After this, it was proposed that intra-tumoural removal of Treg cells should lead to increased anti-tumour immunity, and various attempts have been made to target Tregs lymphocytes as anti-cancer therapy, with different results (Shimizu *et al*, 1999; Yu *et al*, 2005; Hegmans *et al*, 2006; Needham *et al*., 2006). The role of IL-2 in mediating Treg cell homeostasis is still unclear, although evidence has suggested a role of IL-2 in promoting the development and suppressive function of Treg cells (Turka and Walsh, 2008). Few studies have explored Treg cells in MPM (DeLong *et al*, 2005; Hegmans *et al*, 2006; Anraku *et al*, 2008). Anraku *et al* (2008) found a small number of Treg cells in MPM. DeLong *et al* (2005) also reported a lower number of these cells in the pleural effusions from seven MPM patients, comparing with those from lung or breast cancer patients. On the contrary, Hegmans *et al* (2006) showed a significant numbers of Treg cells in biopsies from MPM patients, although a small number of cases (only four cases) were analysed. In our study, we observed a significantly higher number of positive Treg cells in the group of IL-2-treated patients compared with untreated patients, confirming the expansion of the Treg subset observed in patients with melanoma and renal carcinoma who underwent IL-2 therapy (Ahmadzadeh and Rosenberg, 2006). Along these lines, the authors showed that the Treg cell population expanded *in vitro* after IL-2 administration, showing a strong suppressive function of the anti-tumour immune response. These findings suggest that depletion of the Treg subset lymphocytes may enhance the ability of IL-2 to boost host immunity against cancer.

Several studies have correlated IL-2 therapy with angiogenesis. They show that CD8 lymphocytes seem to be required for reducing tumour-associated vascularity (Sakkoula *et al*, 1997; Jackaman *et al*, 2003). In light of these studies, we have also evaluated the MVC and VEGF expression. MVC was significantly lower in IL-2-treated patients compared with untreated patients. In the same way, VEGF expression was lower in IL-2-treated patients, but the difference with untreated patients was not significant. Our results support the hypothesis for a role of IL-2 in inhibiting blood vessels in MPM patients, suggesting a supplementary anti-cancer mechanism that is mediated by IL-2 in this type of tumour.

In our study, we found that non-epithelioid subtypes showed a significantly higher MVC than those with epithelioid histology and this finding could be explained by considering that tumours with sarcomatoid differentiation have more aggressive behaviour and poorest prognosis (Travis *et al*, 2004).

In this study, we confirmed that tryptase-positive MCs predict a better clinical outcome in IL-2-treated patients with MPM (Alì *et al*, 2009). In the same treatment group, we observed that Treg lymphocytes had a negative impact on the prognosis of patients. As far as we know, this is the first study in which a statistically significant correlation was found between Treg lymphocytes and prognosis in MPM. Anraku *et al* (2008) found that Treg cells were negatively correlated with prognosis, but the finding was not statistically significant. They also showed that high levels of CD8+ lymphocytes were associated with a better prognosis and with a lower incidence of mediastinal lymph node metastasis. In our study, we observed the same correlation of CD8+ lymphocytes with prognosis in MPM patients treated with IL-2, although it was not statistically significant.

Interestingly, when counts of tryptase-positive MCs and Treg cells are combined, the outcome of patients can be predicted more effectively, confirming the close relationship between these two immunological parameters (Lu *et al*, 2006; Ju *et al*, 2009). Therefore, tryptase-positive MCs and Treg lymphocytes are promising prognostic parameters that could be used jointly to identify groups of patients with distinct outcomes.

In conclusion, this study indicates that treatment of MPM with intra-pleural IL-2 leads, on one hand to an increase in tryptase-positive MCs, and in CD8+ and Foxp3+ lymphocytes, and on the other hand to an inhibition in tumour-associated vascularity.

These findings give new insights into the complex anti-tumoural mechanisms mediated by IL-2 in malignant mesotheliomas, which are useful if IL-2 is to be successfully used in the clinical setting. Further studies are required to confirm the functional properties of the effector, or regulatory phenotypes, as the anti-cancer effect of IL-2 could be related to the correct balance between these two types of immune cells.

Moreover, we propose that counts of tryptase-positive MCs and Treg lymphocytes may be strong predictors of outcome of MPM patients treated with IL-2. These findings underline the importance of the immunologic analysis in the prognostic and therapeutic approach in patients with pleural mesothelioma. New, tailored immunotherapeutic trials in selected patients may be an effective strategy to improve current regimens and prolong survival of this very aggressive and fatal tumour.

## Figures and Tables

**Table 1:** Patients’ characteristics: untreatment and treatment related

**Clinicopathologic characteristics**	**IL-2-treated patients (*N*=60) *n*. cases (%)**	**Untreated patients (*N*=33) *n*. cases (%)**
*Age*		
Range	41–77 years	54–81 years
Median	62.5 years	70.0 years
		
*Gender*		
Male	51 (85%)	26 (78.8%)
Female	9 (15%)	7 (21.2%)
		
*Histologic subtypes*		
Epithelioid	46 (76.7%)	25 (75.8%)
Biphasic	9 (15.0%)	3 (9.0%)
Sarcomatoid	5 (8.3%)	5 (15.2%)
		
*IMIG stage*		
IB	1 (1.7%)	
II	15 (25.0%)	16 (48.5%)
III	44 (73.3%)	17 (51.5%)
		
*ECOG performance status*		
0	8 (13.3%)	6 (18.2%)
1	34 (56.7%)	20 (60.6%)
2	18 (30.0%)	7 (21.2%)
		
*Recurrence (valid n 60)*		
Yes	41	
Local	33	
Local+sistemic	7	
Sistemic	1	
No	19	
		
*Overall survival (valid n 60)*		
Range	2–60 months	
Median	13 months	
		
*Time to progression (valid n 45)*		
Range	1–57 months	
Median	12 months	

Abbreviations: IMIG=International Mesothelioma interest Group; ECOG=Eastern Cooperative Oncology Group.

**Table 2:** Correlations between immunological and angiogenetic parameters in the IL-2-treated patients’ group and in the untreated patients’ group

**Immunological and angiogenetic parameters (mean±s.d.)**	**IL-2 treatment**	**No IL-2 treatment**	***P*-value**
Tryptase	26.18±17.70	19.32±9.05	**0.04**
Chymase	4.06±5.50	3.40±5.80	0.60
Foxp3	11.80±12.25	6.12±9.90	**0.02**
CD4	60.33±40.38	47.42±35.00	0.13
CD8	90.83±30.64	67.11±46.57	**0.004**
CD34	62.12±22.13	147.70±55.09	**0.0001**
VEGF	33.70±30.95	38.18±35.04	0.64

Abbreviation: IL=interleukin. Bold values indicate *P*<0.05.

**Table 3:** Association of immunological and angiogenetic variables with clinicopathologic parameters

**Features**	**Tryptase MCs**	**Chymase MCs**	**CD8**	**CD4**	**Foxp3**	**MVC**	**VEGF**
*P-values*							
Age	0.77	0.74	0.09	0.63	0.87	0.09	0.97
Gender	0.07	0.98	0.07	0.54	0.14	0.58	0.06
IMIG stage	0.35	0.46	0.06	0.52	0.08	0.06	0.48
Histology	0.98	0.78	0.06	0.96	0.06	**0.0047**	0.08
PS	0.85	0.26	0.94	0.73	0.07	0.99	0.83

Abbreviations: IMIG=International Mesothelioma Interest Group; PS=performance status; MCs=mast cells; MVC=microvessel count. Bold values indicate *P*<0.05.

**Table 4:** Univariate analysis of overall survival and time to progression in MPM patients treated with IL-2

**Features**	**No. of patients**	**Overall survival *P*-value**	**Time to progression *P*-value**
*Age (41–77 mean 61.8) median 62.5*			
⩽62	30		
>62	30	NS	NS
			
*Gender*			
Male	51	NS	NS
Female	9		
			
*IMIG stage*			
IB+II	1+14	NS	NS
III	42		
			
*Histologic subtypes*			
Epithelioid	46		
Biphasic	9	**0.02**	**0.02**
Sarcomatoid	5		
			
*ECOG performance status*			
0	8		
1	32	NS	NS
2	17		
			
*Tryptase MCs*			
Low	16	**0.02**	**0.01**
High	44		
			
*Chymase MCs*			
Low	33	NS	NS
High	27		
			
*MVC (CD34)*			
Low	30	NS	NS
High	30		
			
*FoxP3*			
Low	29	**0.000002**	**0.0039**
High	29		
			
*CD4*			
Low	24	NS	NS
High	24		
			
*CD8*			
Low	29	NS	NS
High	28		
			
*VEGF*			
Low	28	NS	NS
High	30		

Abbreviations: IMIG=International Mesothelioma Interest Group; ECOG=Eastern Cooperative Oncology Group; MCs=mast cells; MVC=microvessel count; MPM=malignant pleural mesothelioma; NS=not significant. Bold values indicate *P*<0.05.

**Table 5:** Multivariate analysis of overall survival according to Cox's model for MPM patients treated with IL-2

**Variables**	***β***	**exp(*β*)**	**s.e. exp(*β*)**	***z***	***P***
Tryptase	−1.054	0.348	0.351	−3.005	**0.02**
Foxp3	1.974	7.201	0.410	4.812	**0.0000015**
Age	−0.002	0.998	0.021	−0.114	0.910
Gender	0.256	1.292	0.565	0.454	0.650
Histology	−0.668	0.513	0.699	−0.955	0.340
Stage	1.411	4.098	0.450	3.135	0.067
Performance Status	1.005	2.732	0.623	1.612	0.110

Abbreviation: MPM=malignant pleural mesothelioma. Bold values indicate *P*<0.05.
